# Noncanonical IFN Signaling: Mechanistic Linkage of Genetic and Epigenetic Events

**DOI:** 10.1155/2016/9564814

**Published:** 2016-12-18

**Authors:** Howard M. Johnson, Chulbul M. Ahmed

**Affiliations:** Department of Microbiology and Cell Science, University of Florida, Gainesville, FL 32611-0700, USA

## Abstract

The canonical model of cytokine signaling via the JAK/STAT pathway dominates our view of signal transduction but provides no insight into the significance of the simultaneous presence of activated JAKs and STATs in the nucleus of cells treated with cytokines. Such a mechanistic shortcoming challenges the usefulness of the model in its present form. Focusing on the interferon (IFN) cytokines, we have developed a noncanonical model of IFN signaling that naturally connects activated JAKs and STATs at or near response elements of genes that are activated by the IFNs. Specifically, cells treated with IFN*γ* showed association of activated STAT1*α* and JAK2 at the GAS element of genes activated by IFN*γ*. For IFN*α* treated cells, the association involved activated STAT1*α* and TYK2 JAK kinase at the ISRE promoter. The power of the noncanonical model is that it provides mechanistic insight into specific gene activation at the level of the associated epigenetics, akin to that of steroid/steroid receptor signaling.

## 1. Introduction

The classical or canonical model of signaling by cytokines such as the interferons (IFNs) involves ligand interaction with receptor extracellular domain, followed by “allosteric changes” in the receptor cytoplasmic domain that results in autophosphorylation of the relevant Janus tyrosine kinases (JAKs) and subsequent tyrosine phosphorylation of receptor cytoplasmic domain(s) [1–4]. The climatic event is the association and tyrosine phosphorylation of the appropriate signal transducer and activator of transcription (STATs) factors. The beauty and frailty of the model lies in the simplicity of the STATs being responsible for the specific functional effects attributed to the IFNs. For example, IFN*γ* signaling via a heterodimeric receptor results in the activation of STAT1*α*, by receptor-associated JAK1 and JAK2, to form an asymmetric dimer which undergoes nuclear translocation to specific promoters of genes that are activated by IFN*γ*. In the case of the family of the 15 or more type 1 IFN subtypes, all acting through the same heterodimeric receptor, JAK1 and TYK2 kinases activate STAT1*α* and STAT2 in conjunction with the receptor cytoplasmic domains. STAT1*α* and STAT2 form a trimeric complex with IFN regulatory factor 9 (IRF9), known as ISGF3, followed by nuclear translocation and association with promoters of genes specifically activated by the type I IFNs.

The canonical model of IFN signaling is remarkably lacking in specificity mechanisms, probably attributable to its attractive simplicity and skewed focus on STATs. In the case of IFNs, it has never been shown that activation of the corresponding STATs independent of the IFNs or their receptors has resulted in the induction of an antiviral state. The discovery of a novel member of the type I IFN family, called IFN*τ*, places a particular strain on the canonical model of IFN signaling. Ovine IFN*τ* was originally identified not as an IFN but as a pregnancy recognition hormone in ruminants [5]. It is produced by the conceptus (placenta) of pregnant sheep. Structurally, the amino acid sequence of IFN*τ* shows 30 to 70 percent homology to other type I IFNs [6]. IFN*τ* operates via the same heterodimeric receptor as all the type I IFNs, is as potent antiviral and antiproliferative agent as IFN*α*, and is equally effective in induction of (2′-5′) oligoadenylate synthetase but unlike IFN*α* it is relatively nontoxic at high doses [7].

It is noteworthy that IFN*τ* and IFN*α* had similar specific antiviral activities but IFN*α* is bound to receptor at a 10-fold higher binding affinity [7]. Antibodies to IFN*τ* C-terminus blocked binding of both IFN*τ* and IFN*α* to the receptor but antibodies to IFN*τ* N-terminus only blocked IFN*τ* binding, suggesting that they recognized the receptor similarly at their C-terminus but differently at their N-terminus. The findings suggested that maximal IFN antiviral activity required only fractional occupancy of receptor by the IFNs while toxicity was associated with maximal receptor occupancy. Consistent with similar antiviral activity, IFN*τ* and IFN*α* phosphorylated JAK kinase TYK2 and STAT1 and STAT2 transcription factors similarly, suggesting that phosphorylation of these signal transduction molecules was associated with antiviral activity and not toxicity. The similar and differential effects of these two type I IFNs operating through the same receptor complex are not readily explained by the canonical model of type I IFN signaling. We have discovered, developed, and characterized a noncanonical IFN signal transduction pathway that is remarkably similar to steroid/steroid receptor signaling (reviewed in [8, 9]). It is our contention that the pathway not only provides the mechanism of genetic and epigenetic signaling by IFNs but also provides insight into other cytokine, growth factor, and hormone signaling pathways.

## 2. The Canonical Model

**Figure 1:**
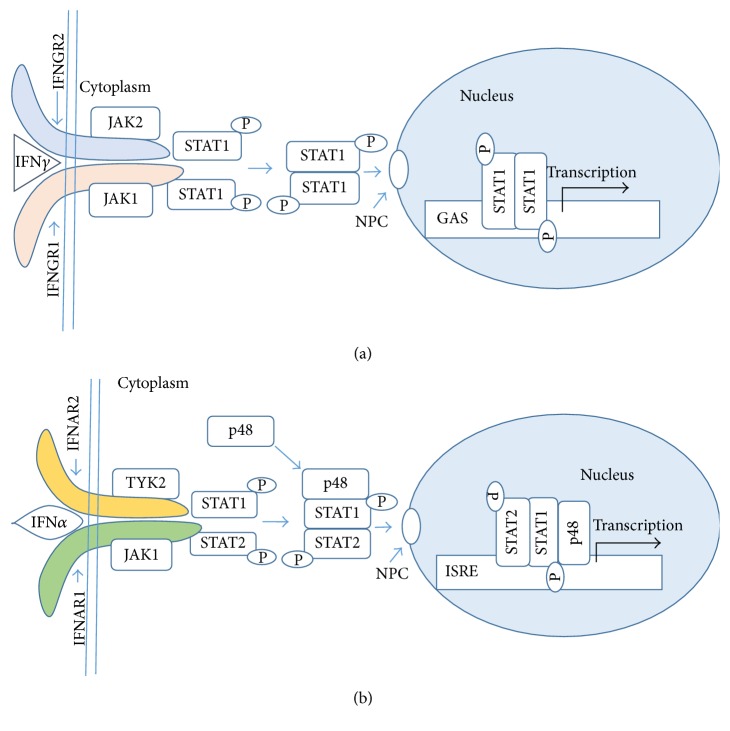
Canonical model of IFN*γ* (a) and IFN*α* (b) signaling. (a) IFN*γ* cross-links the IFNGR1 receptor subunit that results in allosteric changes in receptor cytoplasmic domain, causing the movement of JAK2 from receptor subunit IFNGR2 to IFNGR1. The JAKs autophosphorylate and then phosphorylate IFNGR1 cytoplasmic domain. This results in binding, phosphorylation, and dimer formation of STAT1*α*. The dimeric STAT1*α* dissociates from receptor and undergoes nuclear translocation via an intrinsic NLS for specific gene activation. (b) IFN*α* binding to its receptor subunit initiates a set of events that leads to activation of STAT1*α* and STAT2 and formation of a complex of STATs and p48 (ISGF3) that is translocated to the specific promoter site. This model does not account for the epigenetic events nor does it explain the specificity of cytokine signaling. NPC, nuclear pore complex.

The central players of IFN*γ* signaling via the classical or canonical pathway involve the IFN*γ*, receptor subunits IFNGR1 and IFNGR2, tyrosine kinases JAK1 and JAK2, and STAT1*α* transcription factor [1–4]. Type I IFN signaling involves a type I IFN, receptor subunits IFNAR1 and IFNAR2, tyrosine kinases JAK1 and TYK2, and STAT1 and STAT2 transcription factors.  Figure 1 illustrates the sequence of events from IFN/receptor binding to the presence of activated STATs at the response elements of genes that are specifically activated by the IFNs. Proponents of these classical pathways point out the interaction of the activated STATs with coactivators. STATs in the nucleus, for example, interacting with epigenetic players such as p300 and CREB binding protein (CBP) where CREB means cAMP response element binding protein [2]. p300 and CBP are acetyl-transferases that are involved in chromatin remodeling [10, 11]. IFN also activates other players and pathways, including MAPK (mitogen-activated protein kinase), PI-3K (phosphoinositide 3-kinase), and NF-kB (nuclear factor kB) [2, 12]. These interactions are a sampling of events in IFN activation of STAT transcription factors. All of these and other pathways activated by IFN signaling and STAT activation of the canonical model are generic in the sense that a host of different cytokines, hormones, and growth factors also activate them. There is to date no known specificity-determining orchestration or coordination center identified for any of the STAT or non-STAT aspects of the canonical model. So, identification of these IFN induced events is simply in agreement with that which is known to occur with a plethora of other cytokines that have functions different from that of IFNs. It does not tell us mechanistically why IFNs specifically do what they do.

The report of activated JAK2 in the nucleus is, in our view, a very important event that is a game changer as far as the specific mechanism of cytokine signaling at the level of gene activation including the associated epigenetic events [13]. We briefly describe here and will explore in some depth later why this finding is important in specific gene activation by cytokines. It was shown in 2009 that leukemic cells with gain-of-function mutated JAK2, and JAK2V617F, which is constitutively activated, performs a key epigenetic function in the nucleus [13]. The activated JAK2 phosphorylates histone H3 at tyrosine residue 41 (Y41). Phosphorylated H3Y41, H3pY41, causes the dissociation of heterochromatin protein 1*α* (HP1*α*) from histone H3, resulting in transcription of genes repressed by HP1*α*. Wild-type JAK2 that is activated by growth factors such as platelet-derived growth factor (PDGF) undergo nuclear translocation and similarly phosphorylate histone H3 at Y41, causing dissociation of HP1*α*, resulting in chromatin remodeling for gene activation. The presence of activated JAKs in the nucleus and activated STATs, both activated by the same cytokine, raises the question as to how this fits into events associated with specific gene activation by the cytokine. The canonical JAK/STAT signaling pathway adherents have not addressed this issue. We show that both types I and II IFN activation of TYK2 and JAK2, respectively, via the noncanonical JAK/STAT pathway provides a mechanism for specific gene activation by these IFNs including the associated epigenetics of H3Y41 phosphorylation.

## 3. IFN***γ*** Receptor Interaction and the Noncanonical Model

There are a number of interesting observations concerning IFN activity that are beyond rational explanation or understanding in the context of the canonical JAK/STAT model of IFN signaling. For example, IFN*γ* has been shown to be capable of functioning intracellularly. Specifically, human IFN*γ* delivered by a liposome vector induced an antitumor effect in murine macrophages, expression of nonsecretable human IFN*γ* in murine fibroblasts induced antiviral activity, and microinjected human IFN*γ* induced Ia antigen expression in murine macrophages [14–16]. These findings are at odds with the well-known species preference of exogenous human IFN*γ* which has no activity on murine cells. These reports suggest that IFN*γ* can induce function via an intracellular mechanism that is not species restricted. The fact that these observations were essentially ignored by the IFN community would suggest that they were considered to be of no significance in the context of the canonical JAK/STAT model of IFN signaling.

The IFN*γ* molecule is an asymmetric dimer and the IFN*γ* receptor consists of a noncovalent linked tetramer consisting of two subunits called IFNGR1 and IFNGR2 [12]. According to the canonical model, IFN*γ* cross-links the IFNGR1 subunit, resulting in allosteric changes to the receptor cytoplasmic domain [12]. This allosteric change initiated from the IFN/receptor extracellular interaction is not specified but merely assumed in the context of the model. The intervening hydrophobic transmembrane sequence of the receptor, separating receptor extracellular and cytoplasmic domain, is similarly ignored as to its role in these allosteric events.

Given the stasis of the canonical model in its lack of predictive appeal, we approached IFN*γ* signaling by first doing binding studies of IFN*γ* and IFNGR1. We questioned whether all relevant ligand effects occurred at the receptor extracellular domain. Specifically, we carried out IFN*γ* bindings to intact soluble receptor subunit IFNGR1 consisting of both extracellular and cytoplasmic domains. Using intact IFN*γ*, overlapping IFN*γ* peptides, and overlapping IFNGR1 extracellular and cytoplasmic peptides along with site specific antibodies, we discovered that the N-terminus of IFN*γ* is bound to IFNGR1 extracellular domain and that the C-terminus of IFN*γ* is bound to receptor cytoplasmic domain [17]. Murine IFN*γ* C-terminus peptide, IFN*γ*(95-132), and the corresponding sequence of human IFN*γ* are bound to residues 253-287 of IFNGR1 cytoplasmic domain. This binding was adjacent to the binding site of JAK2 on IFNGR1 and was specifically blocked by anti-(253-287) specific antibodies in fixed/permeabilized cells [17, 18].

It was observed that when cells were treated with IFN*γ* JAK2 binding shifted from receptor subunit IFNGR2 to IFNGR1, presumably as a result of the allosteric changes referred to above [2, 12]. By comparison, we showed that Sepharose coupled JAK2 (Seph-JAK2) bound our soluble radiolabeled IFNGR1 and that such binding was enhanced by both intact IFN*γ* and its C-terminal peptide, IFN*γ*(95-132), but not by the receptor extracellular domain-binding peptide IFN*γ*(1-39) [18]. The enhanced binding of JAK2 was blocked by the IFNGR1 peptide, IFNGR1(253-287), that corresponded to the IFNGR1 binding site for IFN*γ* C-terminus, showing specificity of enhanced binding. A receptor peptide corresponding to the JAK2 binding site, IFNGR1(283-309), also blocked JAK2 binding, while a peptide to an adjacent site had no effect on enhanced JAK2 binding, providing further evidence of specificity. IFN*γ* C-terminus enhancement of JAK2 binding to IFNGR1 cytoplasmic domain would seem to be consistent with the well-known law of mass action, shifting the equilibrium between IFNGR1 and IFNGR2, rather than by allosteric changes evoked by the canonical JAK/STAT model [18].

Functionally, treatment of murine macrophage cell lines with either murine IFN*γ* C-terminal peptide, IFN*γ*(95-132), or its human counterpart resulted in upregulation of MHC class II molecules and induction of an antiviral state similar to IFN*γ* [19]. The peptides also enhanced binding of JAK2 to IFNGR1 in these cells [18]. The peptides were internalized via pinocytosis by the macrophages and were not effective against nonpinocytotic fibroblasts, presumably because they could not access IFNGR1 cytoplasmic domain. This lack of effect on fibroblasts was overcome by attachment of a palmitate to IFN*γ*(95-132), resulting in cell penetration and induction of an antiviral state and upregulation of MHC class II molecules in fibroblasts [20]. These investigators also showed that cells with IFNGR1 gene knockout were refractive to the effects of the IFN*γ* peptide which was evidence that the peptide acted through receptor subunit IFNGR1.

**Figure 2:**
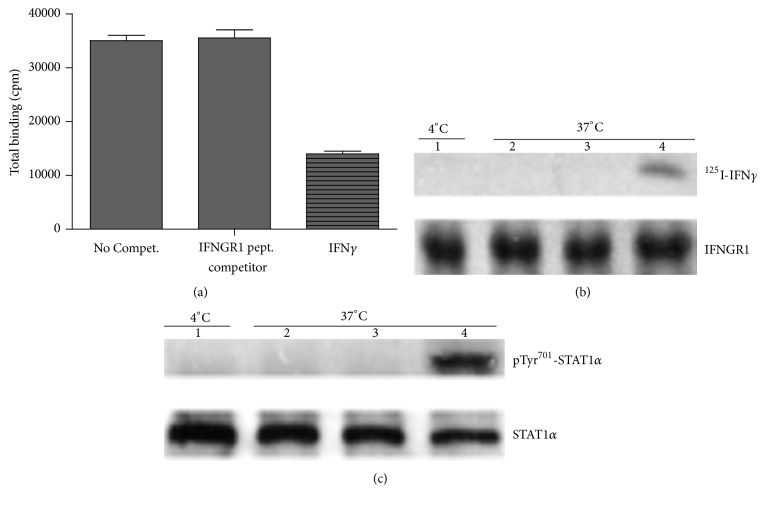
Intracellular presence of peptide IFNGR-1(253-287) inhibits binding to IFNGR of extracellular IFN*γ* and subsequent activation of STAT1*α*. (a) Presence of extracellular peptide IFNGR-1(253-287) did not inhibit binding of ^125^I-IFN*γ* to P388D1 cells at the concentrations to be used in subsequent experiments. Unlabeled murine IFN*γ* or peptide IFNGR-1(253-287), as indicated, was added at a final concentration of 1 *μ*M to P388D1 cells at 4°C along with 10 nM of ^125^I-IFN*γ*, and cells were incubated at 4°C for 30 minutes. Control cells were incubated with ^125^I-IFN*γ* in the absence of any competitor. Cells were then washed and bound IFN*γ* determined. Samples were run in triplicate and values plotted as mean ± s.d. (b) Intracellular accumulation of peptide IFNGR-1(253-287) in P388D1 cells by pinocytosis was accomplished by incubating cells with either 25 *μ*M (lane 2) or 50 *μ*M (lane 3) of peptide at 37°C for 1 hour. Cells used in lanes 1 and 4 did not receive any peptide. Cells were then washed at room temperature to remove extracellular peptide and then incubated with ^125^I-IFN*γ* (10 nM) along with 1 *μ*M of IFNGR-1 peptide for 5 minutes at 37°C. Control cells (lane 1) were washed in ice-cold medium and then incubated with ^125^I-IFN*γ* at 4°C without peptide. After ^125^I-IFN*γ* incubation, all cells were washed at 4°C and then acid-washed at 4°C to remove surface-bound ^125^I-IFN*γ*. Cells were then lysed and immunoprecipitated with antibodies to IFNGR-1. After Western transfer of immunoprecipitates to nitrocellulose membranes, ^125^I-IFN*γ* associated with IFNGR-1 was detected by autoradiography. Total IFNGR-1 immunoprecipitated was followed by immunodetection with IFNGR-1 antibodies (lower panel). (c) Conditions are the same as in (b), except that lysates were immunoprecipitated with STAT1*α* antibodies and tyrosine phosphorylation of immunoprecipitated STAT1*α* was followed by immunodetection with antibodies specific for Tyr701-phosphorylated STAT1*α*. Total immunoprecipitated STAT1*α* was followed by reprobing blots with antibodies to STAT1*α* (lower panel).

The challenge was to show that IFN*γ* interaction with receptor on intact cells finds its way to the IFNGR1 cytoplasm binding site, as per IFNGR1(253-287) peptide, during the process of endocytosis [21]. First, specific binding to a murine cell line (P388D1) was established using radiolabeled murine IFN*γ*, ^125^I-IFN*γ*. Binding was carried out at 4°C to prevent endocytosis. Unlabeled IFN*γ* blocked ^125^I-IFN*γ* binding by competing for receptor while IFNGR1(253-287) that corresponds to the IFNGR1 cytoplasmic binding site for IFN*γ* in soluble receptor binding had no effect on extracellular receptor binding (Figure 2(a)). To assess intracellular binding to the sequence 253-287 of IFNGR1, P388D1 cells were incubated at 37°C to facilitate intracellular loading of the IFNGR1(253-287) peptide. Cells were then incubated at 37°C for a short period with ^125^I–IFN*γ* after which they were lowered to 4°C and surface ^125^I-IFN*γ* was removed by acid treatment. Cell supernatant was treated with antibodies to IFNGR1 and Western blots showed that ^125^I–IFN*γ* was associated with the precipitated IFNGR1 (Figure 2(b)). Importantly, IFNGR1(253-287) loaded cells blocked binding of ^125^I–IFN*γ* to the corresponding site on IFNGR1. An added caveat to this experiment is that blockage of IFN*γ* binding to IFNGR1 cytoplasmic domain resulted in the absence of activation of STAT1*α* as assessed by phosphorylation of tyrosine 701 by JAK2 (Figure 2(c)). Taking together, these binding studies show that IFN*γ* binds extracellular receptor domain and traverses to the cytoplasmic domain of IFNGR1 which is coupled to STAT1*α* activation.

Historically, the smallpox virus has been responsible for billions of deaths and has been estimated to have wiped out as many as 90% of the South American population as a result of European introduction of the virus [22]. A central reason for the potent virulence is probably due to the remarkable refractiveness of the virus to IFNs as a result of the induction of IFN decoy receptors. The vaccinia virus, for example, codes for secreted, soluble proteins B18R and B8R that are truncated such as to retain only the extracellular, ligand binding domain of the receptor that competes with type I and type II IFN, respectively [23, 24]. The IFN based C-terminus mimetics by comparison are potent inhibitors of vaccinia virus because they are not recognized by virus decoy receptors [25, 26]. Thus, the decoy receptors can neutralize the intact IFNs and not the C-terminal peptides that are devoid of the domain involved in the extracellular binding of the decoy receptor. It should be noted that these mimetics are the result of the noncanonical pathway of IFN signaling.

## 4. IFN and the Genetics and Epigenetics of Specific Gene Activation

The abovementioned scenario with IFN*γ* and IFN*γ* receptor subunit IFNGR1 is applicable with variations to type I IFN signaling system. In IFN*γ* signaling, the receptor subunit IFNGR2 remains on the plasma membrane during endocytosis, while IFNGR1 is endocytosed with IFN*γ* [27, 28]. For a type I IFN like IFN*α*, both receptor subunits IFNAR1 and IFNAR2 are endocytosed with the IFN [29]. In this section, we address issues of complex formation in the cytoplasm and movement to specific genes in the nucleus.

The observation that IFN*γ* translocates to the nucleus in receptor-expressing cells with kinetics similar to those of activated STAT1*α* is not considered in the context of the canonical model of JAK/STAT signaling, perhaps because of the central role ascribed to STAT (reviewed in [8, 9]). However, such observations are of potential importance in the context of the noncanonical model of IFN*γ* signaling. We showed that IFN*γ* nuclear translocation was driven by a polycationic nuclear localization sequence (NLS), ^126^RKRKRSR, in its C-terminus that is similar to that of the prototypical SV40 large tumor antigen (T-ag) NLS(PKKKRKV) [30]. Mutations of the IFN*γ* NLS resulted in loss of biological activity which was restored by T-ag prototypical NLS [31]. Efficient nuclear transport via polycationic NLSs involves high affinity recognition by members of the importin (IMP) superfamily of nuclear transport molecules [32–34]. IFN*γ* is actively transported by the heterodimeric IMP*α*/*β* complex in the cytoplasm where IMP*α* binds the IFN*γ* NLS and IMP*β* mediates the interaction with the nuclear pore and Ran, with ATP/GTP as energy source [32]. The IMP association of IFN*γ* was established by immunoprecipitation with antibody to IMP*α* (anti-NPI-1) and Western blotting [31]. Related to this, T-ag which binds to IMP*α* competitively inhibited IFN*γ* function in cells [30].

We showed above that endocytosed IFN*γ* interacted with the cytoplasmic domain of receptor subunit IFNGR1 at residues 253-287 which is adjacent to JAK2 binding site. Immunofluorescent confocal microscopy and cell fractionation studies showed that receptor in lipid microdomains on the cell surface played an important role in the endocytosis and that receptor subunit IFNGR2 did not undergo endocytosis but remained on the cell surface [27, 28]. The function of IFNGR2 may be to serve as reservoir for JAK2 that binds to IFNGR1 with higher affinity as a result of IFN*γ* binding [18].

**Figure 3:**
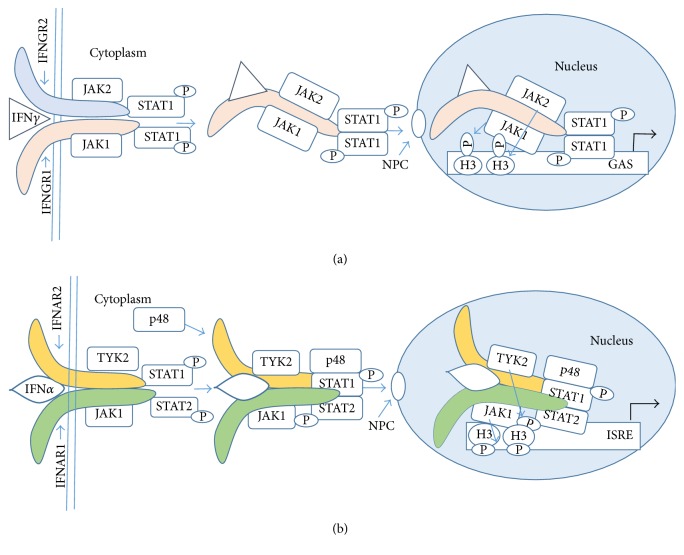
The noncanonical model of IFN*γ* (a) and IFN*α* (b) signaling. (a) Binding of IFN*γ* to its receptor extracellular domain is followed by movement to IFNGR1 cytoplasmic domain in conjunction with endocytosis. The cytoplasmic binding increases the affinity of JAK2 for IFNGR1, which is the basis for its movement from IFNGR2 to IFNGR1. This results in autoactivation of the JAKs, phosphorylation of IFNGR1 cytoplasmic domain, and the binding and phosphorylation of STAT1*α* at IFNGR1. The complex of IFN*γ*/IFNGR1/STAT1*α*/JAK1/JAK2 undergoes active nuclear transport where the classic polycationic NLS of IFN*γ* plays a key role for this transport to genes in the nuclei that are specifically activated by IFN*γ*. JAKs are involved in epigenetic events that cause heterochromatin destabilization and promoter activation. Histone H3 phosphorylation at Tyr 41 by JAK1 and JAK2, indicated by arrows, is a key epigenetic event in IFN*γ* gene activation. (b) IFN*α* signaling involves the endocytosis of IFN*α* and both of its receptor subunits. This complex binds to p48 to generate interferon stimulated gene factor 3 (ISGF3). IFN*α*/IFNAR1/IFNAR2/JAK1/TYK2/p48 are then translocated to the promoter ISRE, where JAK1 and TYK2 are involved in phosphorylation of histone H3. GAS, IFN gamma activated sequence; H3, histone H3; ISRE, IFN sensitive response element; NPC, nuclear pore complex.

Given the central emphasis that has been placed on STATs in specific gene activation by cytokines, it seems reasonable in the case of IFN*γ* to determine what proteins are associated with STAT1*α* at the GAS element in genes activated by this IFN. Accordingly, we used the combination of immunoprecipitation with Western blotting, nuclear confocal immunofluorescence, chromatin immunoprecipitation (ChIP) followed by PCR, and other focused techniques to show that IFN*γ*, IFNGR1, JAK1 and JAK2, and STAT1*α* were all present at the GAS element of genes activated by IFN*γ*. We initially focused on the role of the IFN*γ* NLS in translocation of IFNGR1 into the nucleus [8, 9, 31, 35]. We established that cells treated with IFN*γ* or the internalized IFN*γ* C-terminus peptide IFN*γ*(95-132) resulted in IFNGR1 translocation to the nucleus and that the IFN*γ* NLS was required [8, 9, 31, 35]. We next showed that activated STAT1*α* (pSTAT1*α*) and the activated JAKs, pJAK1 and pJAK2, also required the NLS of IFN*γ* for nuclear translocation, all as a complex of IFN*γ*/IFNGR1/pSTAT1*α*/pJAK1/pJAK2 [8, 9, 31, 35]. We showed that the complex played an essential role in the coordinated events of specific gene activation and the associated epigenetics. Thus, STAT1*α* is but one of the collection of key players in specific gene activation by cytokines such as IFN*γ*. See Figure 3(a) for an illustration of the IFN*γ* events.

We similarly examined type I IFN system for noncanonical signaling. Using the same approach with particular use of ChIP followed by PCR, immunoprecipitations, and confocal microscopy, we showed the association of pSTAT1*α*, IFNAR1, IFNAR2, and TYK2 with the ISRE element of the oligoadenylate synthetase 1 (OAS1) promoter in IFN*α*2 treated cells [29]. Such association was not shown at the *β*-actin promoter after IFN treatment as type I IFN does not activate the *β*-actin gene. See Figure 3(b) for an illustration of the IFN*α* events.

As indicated earlier, both wild-type and gain-of-function mutated JAK2 were shown to be present in the nucleus [13]. Specifically, constitutively activated JAK2V617F was shown to phosphorylate histone H3 on tyrosine 41 (H3pY41) which caused dissociation of the inhibitor HP1*α* from H3. The key result of this was chromatin remodeling to euchromatin which led to gene activation. JAK2V617F mutation is associated with particular hematologic disorders, suggesting that the epigenetic effect involves interaction with the relevant hematological receptor. It is our view that the power of a model is that it both explains and predicts mechanisms. In this regard, it was shown that JAK2V617F association with a homodimeric type I cytokine receptor, the erythropoietin receptor (EpoR), the thrombopoietin receptor, or the granulocyte colony-stimulating factor receptor, was necessary for the induction of the transforming leukemic phenotype [36, 37]. The question of whether receptor/JAK2V617F complexes were present at the promoters of genes that were activated in cancers caused by or associated with JAK2V617F was not addressed. It is our view that hematopoietic receptor activation of JAK2V617F in the cytoplasm is not sufficient to induce the H3pY41 phosphorylation specifically at the genes that are activated by JAKV617F as we are not aware that JAK2V617F possesses such intrinsic properties. Thus, in addition to activation of JAK2V617F, the receptor may also colocalize with the kinase at the specific promoter in the nucleus. These results with mammalian systems were preceded by similar observations in a Drosophila model of hematopoietic tumors, including the suppressive effects of Drosophila HPI on the mutant JAK in conjunction with inhibition of tumor [38]. Importantly, it provides an explanation for the phenotypes of these JAK2V617F associated cancers.

Perhaps the most intriguing finding of our noncanonical approach to IFN signaling was the association of H3pY41 with nuclear pJAK2 and pSTAT1*α* at the IRF-1 promoter in cells treated with IFN*γ* [35], and the association of H3pY41 with nuclear TYK2 and pSTAT1 at the OAS1 promoter in cells treated with IFN*α* [29]. Genes such as *β*-actin, which are not activated by IFNs, were negative for the relevant JAKs and STATs [29, 35]. The fact that IFN*α*-associated TYK2 can phosphorylate Y41 on H3 is evidence that the phosphorylation is not restricted to JAK2. In this regard, the mutated JAKV617F appears to be a special case of a more general process. The key is not just the particular JAK but also the particular cytokine, growth factor, or hormone that is the activator of the JAK.

The question arises as to the significance of JAK induction of H3pY41 to nucleosome transient unwrapping so that factors such as the complexes of our noncanonical studies can become involved in DNA transcription. Specifically, it was shown that H3pY41 increased nucleosome unwrapping and access to transcription factor binding by severalfold [39, 40]. H3 at lysine 56, H3K56, is located at the same DNA-histone interface as H3Y41. Acetylated H3K56, H3K56ac, similarly increased nucleosome unwrapping. However, the combination of H3pY41 and H3K56ac had a multiplicative effect and increased unwrapping by 17-fold. It was concluded that the combination of phosphorylation with acetylation significantly increased DNA accessibility to regulatory transcription complexes.

The movement of membrane receptors to the nucleus following endocytosis is not a rare anomaly just limited to some odd IFN result. On the contrary, there are a plethora of plasma membrane receptors that translocate to the nucleus following ligand/receptor interaction. Polycationic NLSs are virtually ubiquitous in cytokine receptors, ligands, or both [41]. Most of the membrane receptors that traffic to the nucleus tend to signal via the JAK/STAT pathway [41]. Receptor tyrosine kinases (RTKs) such as epidermal growth factor receptor (EGFR), fibroblast growth factor receptor (FGFR), vascular endothelial growth factor (VEGFR), growth hormone receptor (GHR), and insulin receptor are well-known growth factors that have been shown to similarly undergo nuclear translocation with ligand [42, 43]. G protein-coupled receptors (GPCR) involving peptide ligands such as angiotensin also have been found in the nucleus [44]. EGFR was the first RTK to be shown to be a cotranscription factor [45].

Considerable work has also been done on the mechanism of nuclear transport of membrane receptors. We showed that internalized IFN*γ* is bound to the cytoplasmic domain of IFNGR1 but the challenge is to decipher the mechanism (Figure 2). We have addressed this issue in part where we showed that the presence of IFNGR1 and IFNGR2 in the lipid microdomain was central to the endocytosis that is linked to the noncanonical signaling pathway [28]. The cytoplasmic domain of IFNGR1 in the endocytic vesicle is exposed to the cytoplasm as intracellular injected antibodies specific to IFN*γ* C-terminus blocked nuclear translocation as well as STAT1*α* activation while the antibodies had no effect on nuclear accumulation of STAT1*α* in cells treated with IFN*α* [31]. Retrograde trafficking of the receptor tyrosine kinase EGFR from the cell surface into the nucleus has been studied extensively [46, 47]. Following EGF induced endocytosis, the endocytic vesicles with the EGFR fuse with early endosomes which traffic to golgi. Retrograde trafficking was blocked by brefeldin A or dominant negative mutants of the small GTPaseARF (ADP-ribosylation factor). Both treatments resulted in disassembly of the COPI (coat protein complex I) which was interpreted as COPI regulation of retrograde vesicular trafficking of EGFR from the Golgi to the ER (endoplasmic endothelium) [46]. The Sec61 translocon was shown to be required for trafficking of EGFR from the ER into the nucleus [47]. Epigenetically, EGFR has been shown to modulate DNA synthesis and repair through phosphorylation of tyrosine on histone H4 at residue H4Y72 which is connected to enhanced methylation at H4 K20 [43]. Thus, there are specific mechanistic data on a key epigenetic event associated with activation of EGFR. Mechanisms of nuclear translocation and some epigenetic effects have similarly been reported for other RTKs [48, 49].

## 5. Conclusions

Receptor-associated tyrosine kinases such as the JAKs and RTKs such as EGFR in the nucleus are probably key players in normal and abnormal cellular activity. Our noncanonical model of IFN signaling where JAKs, receptors, and STATs are physically linked significantly demystifies genetic and epigenetic aspects of cytokine signaling. JAK2V617F associated hematopoietic cancers are much better understood in the context of JAK2V617F linkage with receptors such as EpoR for both activation and specific function in the nucleus. The same pertains to RTKs where the kinase is part of the receptor. The noncanonical signaling model should thus provide insight into regulation of both homeostatic and nonhomeostatic cellular processes.
